# *GmGIF5* Promotes Cell Expansion by Negatively Regulating Cell Wall Modification

**DOI:** 10.3390/ijms26020492

**Published:** 2025-01-09

**Authors:** Hongmiao Jin, Shiyu Gao, Yingtao Xia, Meiling Hu, Yueping Zheng, Shenhua Ye, Yihua Zhan, Mengyuan Yan, Hongbo Liu, Yi Gan, Zhifu Zheng, Tian Pan

**Affiliations:** The Key Laboratory for Quality Improvement of Agricultural Products of Zhejiang Province, College of Advanced Agricultural Sciences, Zhejiang A&F University, Hangzhou 311300, China; jhm@stu.zafu.edu.cn (H.J.); 2021601022044@stu.zafu.edu.cn (S.G.); 2857652488@stu.zafu.edu.cn (Y.X.); hml@stu.zafu.edu.cn (M.H.); zhengyp@zafu.edu.cn (Y.Z.); yeshenhua@zafu.edu.cn (S.Y.); yhzhan@zafu.edu.cn (Y.Z.); yanmengyuan@zafu.edu.cn (M.Y.); hbliu@zafu.edu.cn (H.L.); zjuganyi@163.com (Y.G.)

**Keywords:** GRF-INTERACTING FACTORs, soybean, cell expansion, transcription factor, RNA-seq

## Abstract

Soybean is an important and versatile crop worldwide. Enhancing soybean architecture offers a potential method to increase yield. Plant-specific transcription factors play a crucial, yet often unnoticed, role in regulating plant growth and development. *GRF-INTERACTING FACTOR (GIF)* genes are plant-specific transcription factors; however, their functions in soybean remain poorly understood. Eight *GmGIF* members were identified in soybean (*Glycine max* L.). Phylogenetic analysis divided the eight GmGIF proteins into three groups. In this study, we focused on the role of *GmGIF5* owing to its high expression level in the meristem. Subcellular localization and transcriptional activity analysis showed that GmGIF5 was localized to the nucleus and has self-transactivation ability. To elucidate the biological function of *GmGIF5*, we constructed transgenic Arabidopsis lines overexpressing the gene. Phenotype observations indicated that the overexpression of *GmGIF5* contributed to larger leaves, higher plants, wider stems, and larger seeds. The organs of *GmGIF5* overexpression lines exhibited larger sizes primarily due to an increase in cell size rather than cell number. RNA sequencing was performed to investigate the underlying mechanism for these effects, showing that differentially expressed genes in overexpression lines were mainly enriched in cell wall modification processes. Our study provides new clues for an understanding of the roles of the *GmGIF* family in soybean, which can promote the further application of these genes in genetic breeding.

## 1. Introduction

Soybean (*Glycine max* L.) is a vital crop worldwide with multiple applications. Soybean was first domesticated in China over 5000 years ago, and its cultivation has rapidly expanded since then. This expansion has resulted in a broad distribution range covering the United States, Brazil, Argentina, and India [1]. Soybeans are enriched in oil and protein, offering a nutritious and valuable food source for humans. As a green manure, soybean is also used to maintain fertile soil and feed livestock. Soybean contributes to over one-fourth of the global protein supply for both human consumption and animal feed [1]. The growing human population leads to a higher food demand, which in turn necessitates germplasm resource optimization. Given the significant role of soybeans in societal development, rapidly increasing soybean yield through biotechnological methods is an effective approach to meeting the increasing demand [2]. Therefore, understanding the molecular mechanisms underlying soybean growth and development is of great importance to increasing soybean yield.

Transcription factors (TFs) are crucial in plant growth and development. Recent studies have indicated that some important TFs also regulate yield. Members of the nuclear factor Y-C (NF-YC) TF family are increasingly recognized as key regulators in soybean breeding. *GmNF-YC9* can activate the expression of soybean squalene epoxidase 1 (*GmSQE1*) under both drought and salt conditions to ensure a high content of sterol compounds and improve grain fullness, as well as to enhance yield per plant [3]. MYB is one of the largest plant TF families with primary functions in regulating plant metabolism. The stable overexpression of *GmMYB14* was shown to modulate plant architecture, leading to a high-density yield by increasing the number of pods and seeds, and seed weight per plant [4]. The DNA binding with one finger (Dof) TF family was found to contribute to an increased yield by influencing associated biosynthetic pathways in soybean. Specifically, the overexpression of *GmDof4* and *GmDof11* improved the total fatty acid content and seed size in transgenic Arabidopsis [5]. A non-canonical basic helix–loop–helix (bHLH) TF in wheat, TabHLH489, was identified as a regulator of grain length and weight. Its overexpression resulted in reduced grain length and weight due to the downregulation of genes associated with cell elongation (*TaEXPA4* and *TaEXPA4*) during the early stages of seed development [6]. Moreover, the GROWTH-REGULATING FACTOR (GRF) and GRF-INTERACTING FACTOR (GIF) module has received increased research attention regarding its role in regulating plant cell proliferation and leaf development. In rice (*Oryza sativa*), OsGRF4 interacts with OsGIF1 to regulate grain size [7], and OsGRF6 and OsGRF10 interact with OsGIF1 to activate the expression of rice *JMJD2 FAMILY JMCC GENE 706* (*OsJMJ706*) and *CRINKLY4 RECEPTOR-LIKE KINASE* (*OsCR4*), thus regulating spikelet development [8]. In *Sedum plumbizincicola*, the overexpression of *SpGRF4-SpGIF1* improved transgenic efficiency and accelerated the process of bud regeneration. However, this effect was accompanied by enlarged tissue and abnormal growth [9]. To date, most studies of this module have largely focused on the roles of GRFs, with GIFs considered secondary factors.

The GIF family is a plant-specific TF family found in seed plants. As well-known interacting proteins of GRFs, GIFs were first identified in Arabidopsis [10], with three members reported to date. These can promote the growth of leaves and above-ground organs [11,12]. Overexpressing *AtGIF1* results in the development of larger leaves due to an increase in the number of cells. Mechanistic studies showed that *AtGIF1* expression was directly downregulated by the TPL–KIX–PPD–MYC complex via the G-box, resulting in a decrease in seed size in Arabidopsis [13]. The AN3 TF (encoding *GIF1*) inhibits cell differentiation during the cell proliferation phase [14]. *OsGIF1* positively regulates the size of multiple organs, including the leaf, stem, and grain [15,16]. In addition, *GIF*s participate in controlling quiescent center organization and the meristem, with *gif* mutants displaying an enlarged root meristem and extra root cap layers [11]. Another study showed that *PbGIF1* was induced by cytokinins in parthenocarpous pear fruit, and the overexpression of *PbGIF1* promoted cell proliferation and fruit size in transgenic tomato and pear fruit [17]. According to these previous studies, the *GIF* family plays a crucial role in the process of cell proliferation. However, the function of *GIF* family genes in soybean remains unknown.

In this study, we analyzed the potential functions of *GmGIF5*. The *GmGIF5* gene is highly expressed in meristematic tissues, and its encoded protein is mainly localized in the nucleus. The heterologous expression of *GmGIF5* in Arabidopsis resulted in increased leaf size, plant height, stem width, and seed size. The enlarged organs in *GmGIF5*-overexpressing lines primarily stem from increased cell size rather than expanded cell number. Through combined phenotype observations and an RNA-sequencing (RNA-seq) analysis of the overexpression lines, it is proposed that *GmGIF5* positively regulates cell expansion by influencing genes associated with cell wall modification, offering a new target for improving soybean yield through genetic engineering.

## 2. Results

### 2.1. GmGIF5 Exhibits High Expression in the Shoot Tip

**Figure 1:**
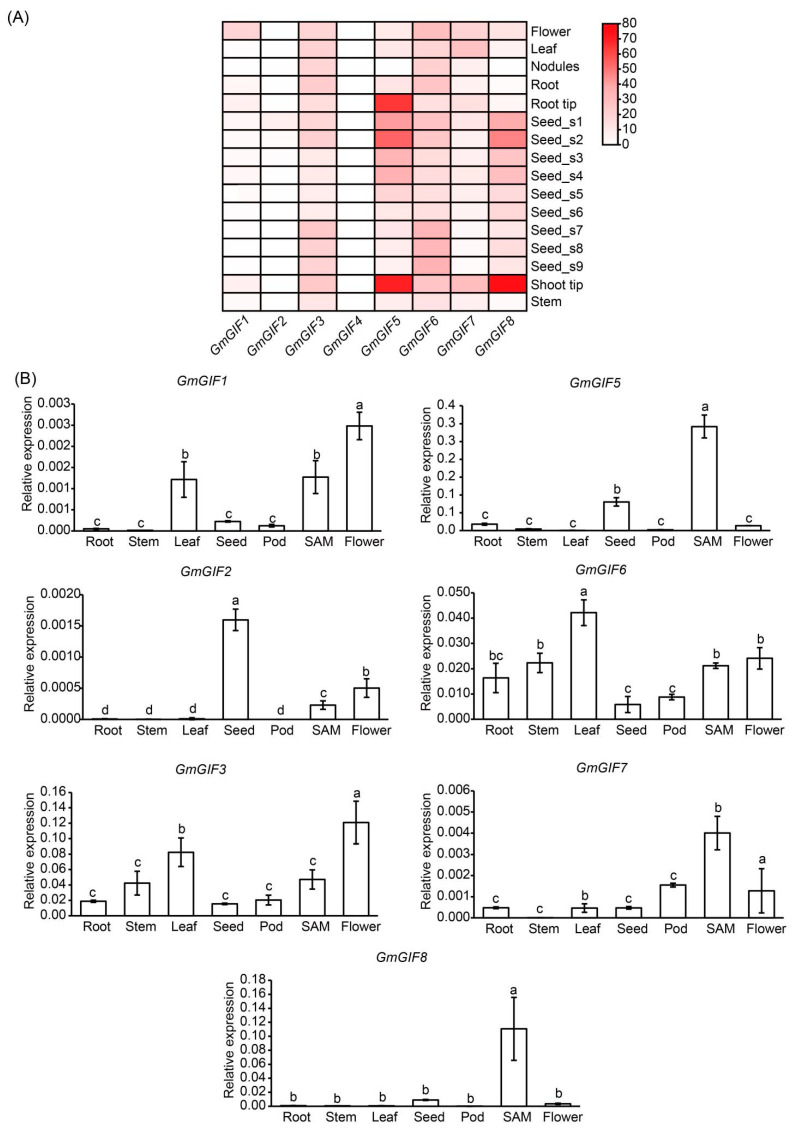
The expression profiles of *GmGIF* genes in soybean tissues. (**A**) The expression patterns of *GmGIF* genes across various tissues were analyzed using transcriptome data, with FPKM values employed to construct this heatmap. Gene IDs of the analyzed genes can be found in Table S1. (**B**) RT-qPCR analysis to reveal *GmGIF* expression patterns in various soybean tissues. The letters of the alphabet represent the results of multiple comparisons when *p* = 0.05.

There are eight *GmGIF* genes in soybean (Table S1) [18]. To understand the phylogenetic relationships of these GmGIF proteins, we extract the GIF protein sequences from *Arabidopsis thaliana*, *Arachis hypogaea*, *Oryza sativa*, *Zea mays*, *Brassica napus*, and *Gossypium hirsutum genome*. According to the phylogenetic tree (Figure S1, Table S2), these eight GmGIF proteins were divided into three groups: GmGIF1, GmGIF5, GmGIF7, and GmGIF8 form Group I; and GmGIF3, GmGIF4, and GmGIF6 form Group II; while Group III contains only GmGIF2. To enhance the understanding of their functions, we analyzed the transcriptional profiles of *GmGIFs* in different soybean tissues (Figure 1A, Table S3). Among the members of Group I, *GmGIF5* showed high expression levels in the root tip, shoot tip, and seed development stages, while *GmGIF8* was mainly highly expressed in the shoot tip and during seed development. The expression pattern of *GmGIF7* was similar to that of *GmGIF5* and *GmGIF8*, but at lower levels, while the expression of *GmGIF1* was low in all tested tissues. Among the members of Group II, *GmGIF3* and *GmGIF6* were expressed in all tissues, whereas the expression of *GmGIF4* was barely detectable. *GmGIF2* from Group III showed weak expression during seed development. Furthermore, RT-qPCR experiments were conducted to validate the RNA-seq analysis results, and the results were largely consistent with the transcriptomic data (Figure 1B). Overall, these results suggest that *GmGIF5* and *GmGIF8* may play a role in soybean growth and early seed development. Given that *GmGIF5* had higher expression levels, we selected this gene for further functional exploration.

### 2.2. Nuclear Protein GmGIF5 Has Self-Transactivation Activity

**Figure 2:**
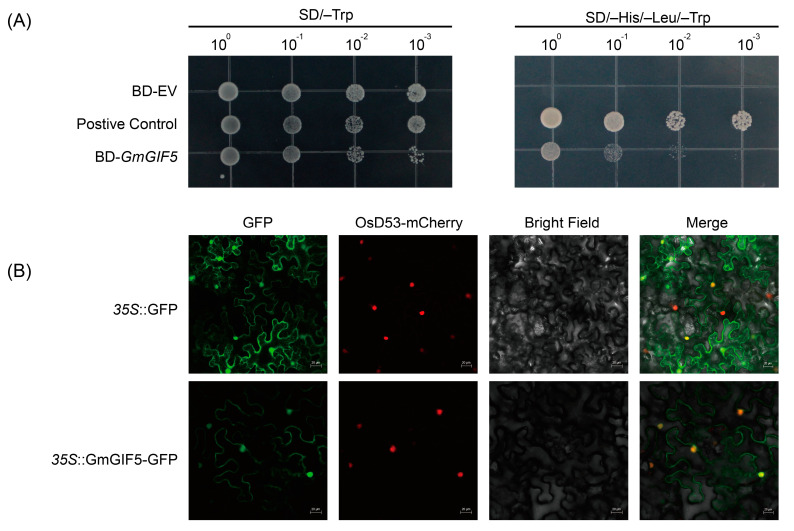
Transcriptional activity and subcellular localization analysis of GmGIF5. (**A**) Self-transactivation assay of *GmGIF5* in yeast. Yeast cells harboring the BD-*GmGIF5*, positive control BD-*GmNAN181* or negative control BD-EV transformation products were grown on an SD/-Trp medium and SD/-His/-Leu/-Trp medium. (**B**) Tobacco leaves were transformed with a plasmid harboring GFP or the GmGIF5–GFP fusion construct. Tobacco leaves transformed with *35S::*GFP served as the control. The detection of fluorescence was performed under a confocal laser-scanning microscope. Scale bar = 20 μm.

The *GIF* gene family is a group of plant transcription factors uniquely found in seed plants. To evaluate whether *GmGIF5* possesses self-activation ability, a *pGBKT7*(*BD*)*-GmGIF5* fused vector was constructed. Yeast assays revealed that both the negative control (BD-EV) and the yeast strains containing BD-GmGIF5 grew normally on an SD/-Trp medium. However, the negative control strain failed to grow on the SD/-His/-Leu/-Trp medium, whereas the yeast cells harboring BD-*GmGIF5* grew normally, indicating that *GmGIF5* has self-activation capability (Figure 2A).

GmGIF5 was predicted to be localized in the nucleus (Table S1). To further validate the intracellular localization of the GmGIF5 protein, we introduced recombinant vectors carrying *35S::GmGIF5-GFP* into tobacco leaf cells via *Agrobacterium*-mediated transient transformation. The fluorescence signal emanating from the control vectors was detected in both the nucleus and the cytoplasm. Additionally, the colocalization signal between GmGIF5-GFP and the nucleus marker (OsD53-mCherry) was captured, thus indicating the nuclear localization of GmGIF5 (Figure 2B). In summary, these experimental results indicate that GmGIF5 not only has self-activation ability but also is localized in the nucleus, consistent with its role as a transcription factor.

### 2.3. Overexpression of GmGIF5 Contributes to Leaf Development

**Figure 3:**
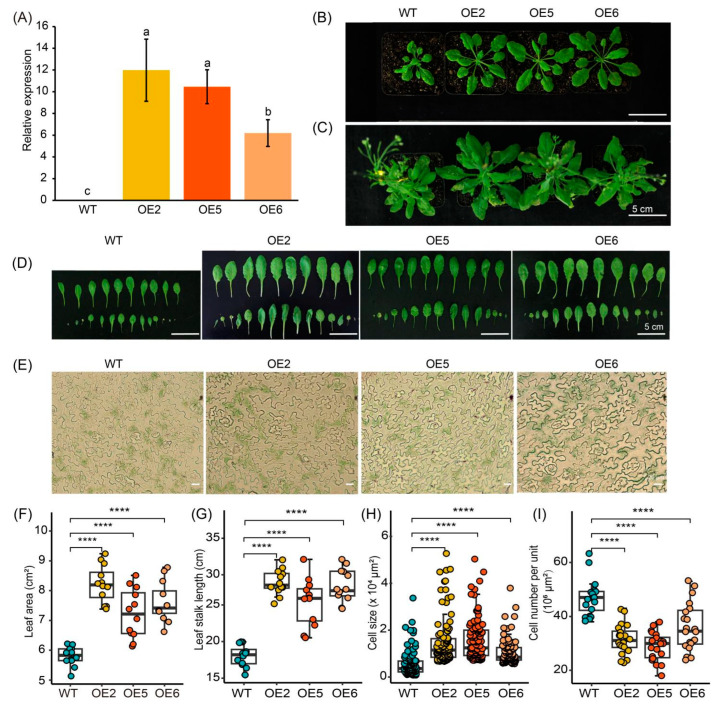
Overexpression of *GmGIF5* promotes leaf development. (**A**) RT-qPCR examination of transgenic Arabidopsis lines. (**B**,**C**) Observation of transgenic Arabidopsis lines at 30 days and 45 days after transplantation. (**D**) Morphological observation of leaves on the entire plant 45 days after transplanting. (**E**–**I**) Measurements of leaf size, petiole length, and epidermal cell size 45 days after transplantation. Boxes represent the median values and the first and third quartiles; whiskers represent the minimum and maximum values. The dots represent single values in boxplots. **** *p* < 0.0001 (Student’s *t*-test). The letters of the alphabet represent the results of multiple comparisons when *p* = 0.05.

To further investigate the functional roles of *GmGIF5* in plant development, we constructed transgenic Arabidopsis lines overexpressing *GmGIF5* driven by the *pUBQ10* promoter. Putative transformants were examined by RT-qPCR, and three positive lines (OE2, OE5, and OE6) were selected for further study (Figure 3A and Figure S2). We observed the growth and development of wild-type (WT) and OE lines and found that while the number of rosette leaves in OE lines was consistent with that in the WT, the leaf size of OE lines was significantly larger compared to that of the WT (Figure 3B–D). Further statistical analysis showed that the projected area of OE line leaves was 1.2 to 1.4 times larger than that of the WT, and the leaf stalk length of OE lines was 1.4 to 1.6 times longer than that of the WT (Figure 3F,G). To understand the mechanism behind the increased leaf size in the OE lines, we examined the leaf epidermis cells of both WT and OE lines under a microscope, calculating cell number and cell size (Figure 3E,H,I). The results showed that the leaf epidermal cell size of the three OE lines was significantly increased compared to that of the WT. Additionally, the number of cells per unit area in OE lines was significantly reduced compared to in the WT. These findings indicate that *GmGIF5* influences leaf development by regulating the size of leaf cells. Overall, these results demonstrate that *GmGIF5* plays a crucial role in leaf growth and development in plants.

### 2.4. Overexpression of GmGIF5 Promotes Stem Development

**Figure 4:**
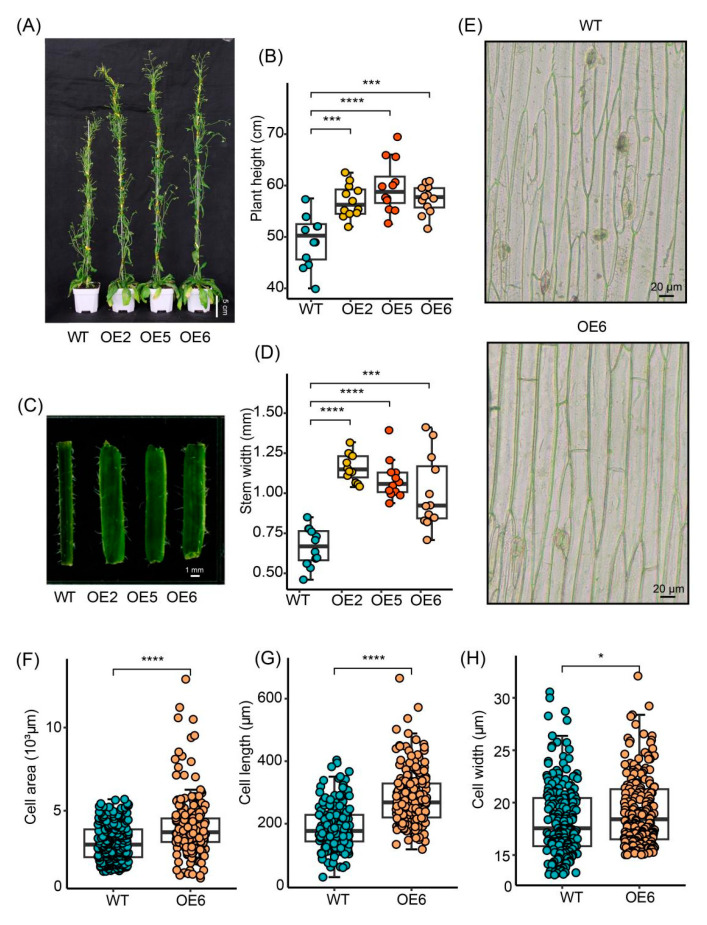
Overexpression of *GmGIF5* enhances stem development. Plant height (**A**,**B**) and stem width (**C**,**D**) of transgenic lines and the wild type (WT) at 75 days after transplantation. (**E**–**H**) Measurement of stem cell size in overexpression lines and wild-type lines. Boxes represent the median values and the first and third quartiles; whiskers represent the minimum and maximum values. The dots represent single values in boxplots. * *p* < 0.05, *** *p* < 0.001, and **** *p* < 0.0001 (Student’s *t*-test).

Since *GmGIF5* is primarily expressed in the stem apex, it likely plays a role in apical growth. We conducted an ongoing analysis of transgenic Arabidopsis lines, and at 75 days post-transplantation, during the productive stage, the average height of *GmGIF5*-overexpressing plants was 1.15 to 1.21 times that of the wild type (Figure 4A,B). Additionally, the stems of the overexpression lines were significantly thicker compared to those of the wild type, with the average stem width of the OE lines being 1.51 to 1.75 times greater than that of the wild type (Figure 4C,D). We hypothesized that cell expansion might be responsible for the increased height and thicker stems in the overexpression lines. A microscopic observation of the epidermis of the main stem, collected 1 cm above the rosette leaf, confirmed this hypothesis (Figure 4E–H and Figure S3). These findings indicate that *GmGIF5* is involved in stem development, and its overexpression in Arabidopsis increases plant height and enhances stem thickness.

### 2.5. GmGIF5 Regulates Seed Size

**Figure 5:**
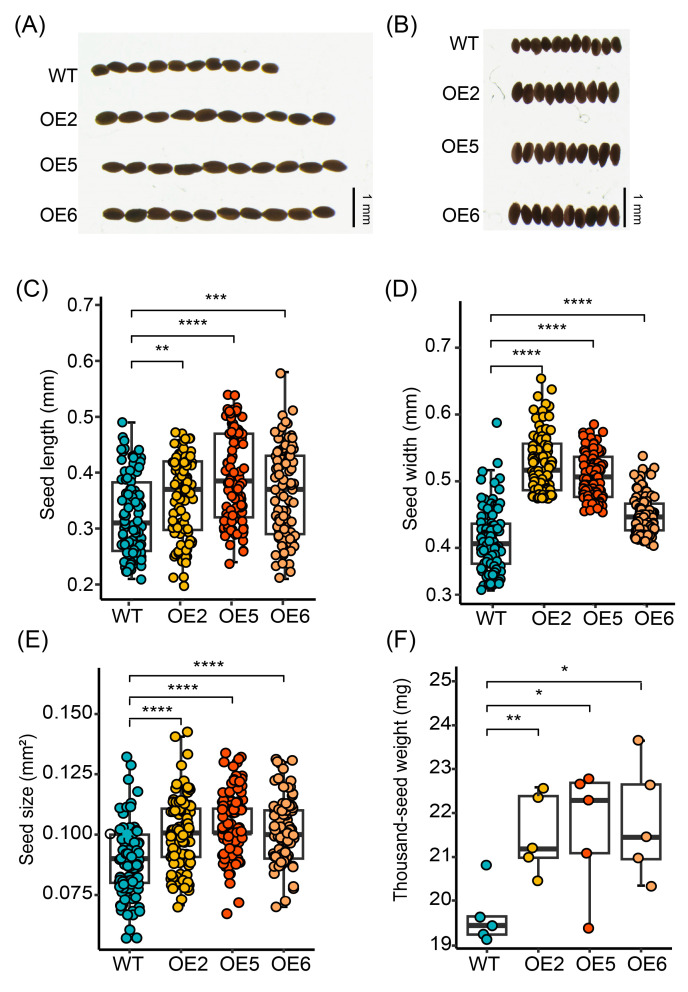
Overexpression of *GmGIF5* affects seed size. (**A**,**B**) Seed phenotype of transgenic lines and the WT at 75 days after transplantation. (**C**–**F**) Measurement of seed length (**C**), seed width (**D**), seed size (**E**), and thousand-seed weight (**F**) in overexpression lines and wild-type lines. Boxes represent the median values and the first and third quartiles; whiskers represent the minimum and maximum values. The dots represent single values in boxplots. * *p* < 0.05, ** *p* < 0.01, *** *p* < 0.001, and **** *p* < 0.0001 (Student’s *t*-test).

Given that the overexpression of *GmGIF5* affected the cell size of leaves and stems, we observed both wild-type and overexpressing line mature seeds to determine whether it also had an impact on seeds. The results showed that the lengths and widths of seeds from the overexpression lines were greater than those of the wild type (Figure 5A,B). Statistical analysis further confirmed that the projected area of the seeds of the three overexpression lines was significantly larger than that of the wild-type (Figure 5C–E). Additionally, the thousand-grain weight of the overexpression lines was also significantly increased compared to that of the wild type (Figure 5D). In summary, *GmGIF5* can promote cell expansion, enhance plant biomass accumulation, and facilitate seed development.

### 2.6. Transcriptomic Analysis of GmGIF5-Overexpressing Arabidopsis

To elucidate how an increased *GmGIF5* level promotes cell expansion, we performed RNA-seq using the WT and OE2 lines. Six libraries were obtained with an average of 4.30 million clean reads. The libraries had an average Q30 rate of 96% and GC content of 45%. A total of 123 genes were screened as DEGs, with only 26 of these genes upregulated in the OE2 line (Figure S4 and Table S4). A principal component analysis and correlation analyses showed that our identification results were plausible (Figure S5).

**Figure 6:**
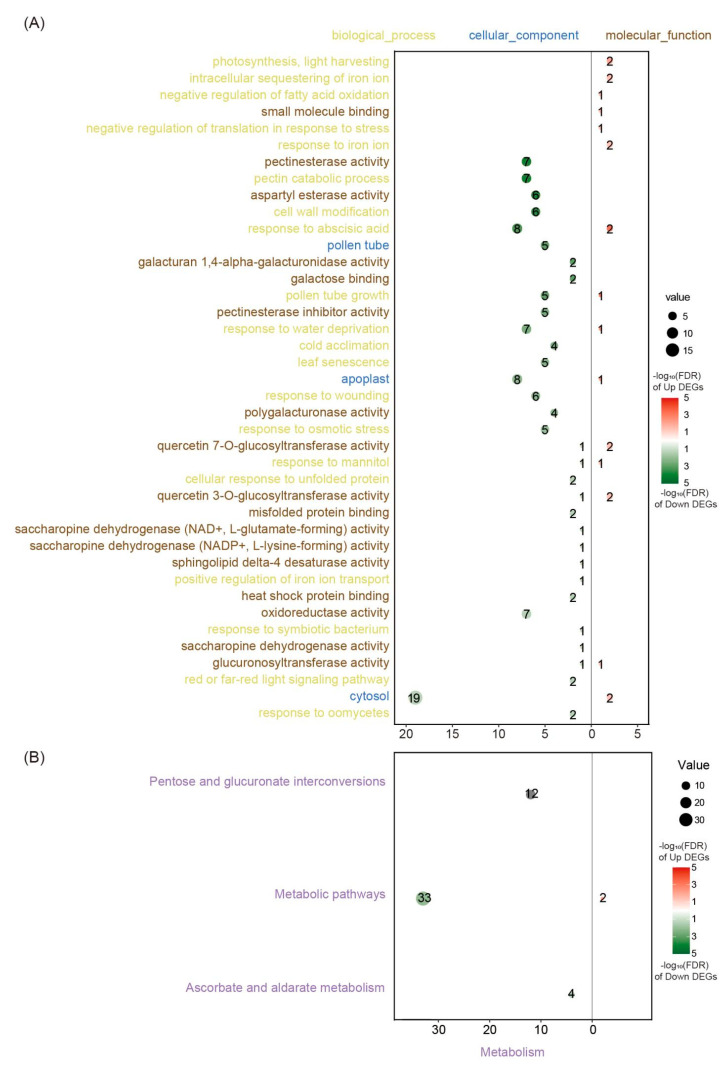
Function enrichment analysis of differentially expressed genes between overexpression 2 (OE2) and wild-type (WT) lines. (**A**) Gene Ontology (GO) and (**B**) Kyoto Encyclopedia of Genes and Genomes (KEGG) pathway enrichment of differentially expressed genes between OE2 and WT lines. Yellow indicates biological process terms, blue indicates cellular component terms, and brown indicates molecular function terms. Purple indicates metabolic pathways. Upregulated genes are shown in red, and downregulated genes are shown in green. The size of the dots reflects the number of genes. The numbers on the dots show the number of enriched genes..

To better understand the functions of *GmGIF*-regulated genes, we performed Gene Ontology (GO) enrichment analysis (Figure 6A, Table S5). The upregulated genes were enriched in photosynthesis, light harvesting (GO:0009765), the intracellular sequestering of iron ion (GO:0006880), and the negative regulation of fatty acid oxidation (GO:0046322), whereas the downregulated genes were enriched in several essential biological pathways, mainly related to the pectin catabolic process (GO:0045490), pectinesterase activity (GO:0030599), pectinesterase inhibitor activity (GO:0046910), and cell wall modification (GO:0042545). The Kyoto Encyclopedia of Genes and Genomes (KEGG) pathway enrichment analysis revealed significant enrichment (false discovery rate < 0.05) in only three pathways: pentose and glucuronate interconversions (ko00040), ascorbate and aldarate metabolism (ko00053), and metabolic pathways (ko01100) (Figure 6B, Table S5).

Plant cell walls consist mainly of cellulose, hemicellulose, and pectin. Pectin interacts with cellulose and hemicellulose to affect the elasticity, hardening, and permeability of the cell wall [19,20]. Based on the results above, the pectin metabolism pathway and the cell wall modification pathway are likely to play crucial roles in the regulation of cell expansion in *GmGIF5* overexpression lines.

**Figure 7:**
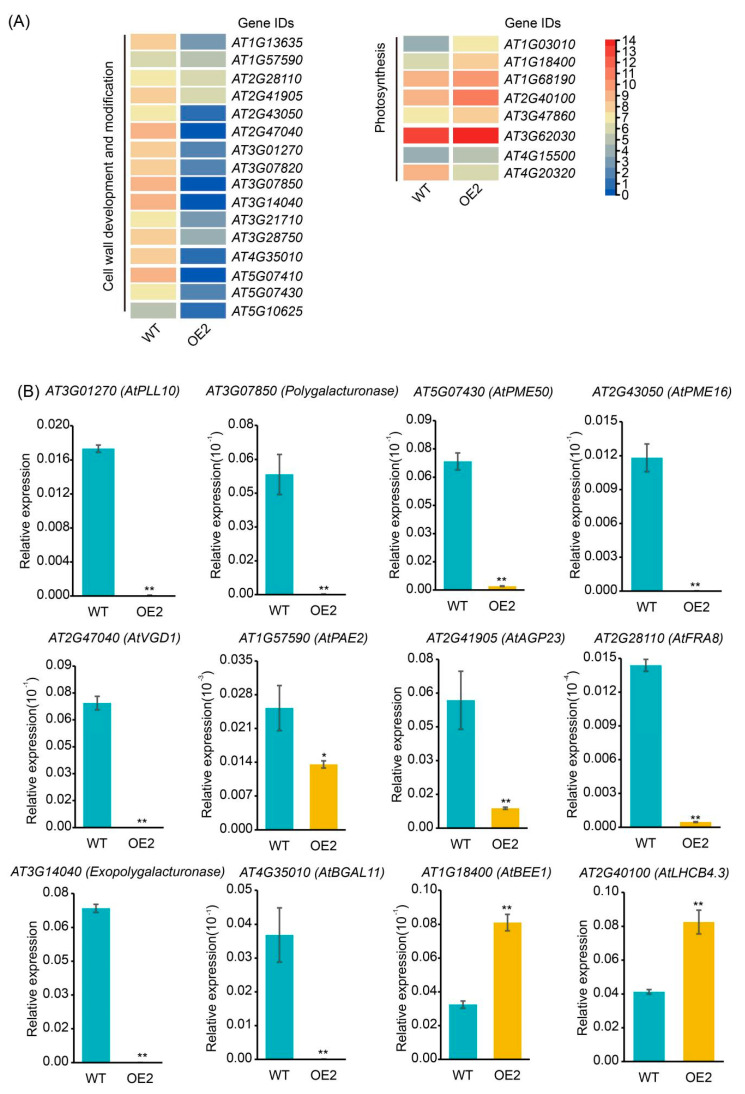
Expression profiles of differentially expressed genes among wild-type (WT) and overexpression 2 (OE2) lines. (**A**) Functional classification of differentially expressed genes and their expression profiles. Log_2_ fold change values are shown along the color scale varying from blue to red; a deeper color indicates a more dramatic change. (**B**) Expression analysis of differentially expressed genes in WT and OE2 lines. * *p* < 0.05, ** *p* < 0.01 (Student’s *t*-test).

In combination with the phenotypic observations of the overexpression (OE) lines, we conducted a further analysis of the RNA-seq data. Interestingly, a significant number of downregulated genes were primarily involved in cell wall modification, particularly in the pectin metabolism modification pathway, while the expression levels of genes related to photosynthesis were upregulated (Figure 7A). As a crucial component of the cell wall, pectin regulates the permeability of the cell wall structure. The degradation and modification enzymes of pectin, such as pectin lyase and pectin methylesterase, are crucial in the structural regulation of plant cell walls [19,20]. We selected several genes for RT-qPCR validation, and the results were consistent with the RNA-seq data (Figure 7B). *AT3G01270* (*PECTATE LYASE-LIKE PROTEIN 10*, *AtPLL10*) genes [21], which encode a pectate lyase-like protein and are reported to participate in pectin degradation and impact cell wall stiffness, were almost unexpressed in the overexpression lines. Meanwhile, *AT2G43050* (*PECTIN METYLESTERASE 16*, *AtPME16*), *AT2G47040* (*VANGUARD1*, *AtVGD1*), and *AT5G07430* (*PECTIN METYLESTERASE 50*, *AtPME50*), which encode pectin methylesterase [22,23], and *AT1G57590* (*PECTIN ACETYLESTERASE 2*, *AtPAE2*), which encodes pectin acetylesterase [24], showed significantly downregulated expression in OE2 compared to the wild type. Additionally, genes encoding other cell wall-related genes, such as *AT3G07850* (polygalacturonase), *AT3G14040* (exopolygalacturonase) [25], *AT2G41905* (*ARABINOGALACTAN PROTEIN 23*, *AtAGP23*) [26], *AT2G28110* (*FRAGILE FIBER8*, *AtFRA8*) [27], and *AT4G35010* (*β-GALACTOSIDASE 11*, *AtBGAL11*) [25], also exhibited significantly downregulated expression in OE2. In summary, the regulation of cell size by *GmGIF5* is achieved through its influence on the expression of cell wall modification genes.

Notably, the expression of *AT1G18400* (*BR-ENHANCED EXPRESSION1*, *AtBEE1*) was upregulated in *GmGIF5*-OE lines, a gene that has been reported to function as a positive regulator in brassinosteroid signaling and photoperiodic flowering [28]. Concurrently, *AT2G40100*, which encodes the light-harvesting complex photosystem II (LHCB4.3), also exhibited significantly upregulated expression in GmGIF5-OE lines. These findings suggest that GmGIF5 may stimulate photosynthesis to promote biomass accumulation.

## 3. Discussion

Soybean has become an increasingly important crop used as oilseed, biodiesel, human food, and animal feed. An increase in soybean yield can help to alleviate the worldwide food shortage crisis. In plants, numerous genes that regulate organ size and shape have been identified. Among them, *CYP78As* play a pivotal role in enhancing organ size [29]. Additionally, transcription factors like Dof and bHLH are significant players in the modulation of plant organ development, making them invaluable targets for genetic engineering in breeding programs. *GIF*s have been reported to regulate cell number and organ size in many plant species, especially seed size and leaf size; however, research on the function of *AtGIF1* homologs in the soybean genome remains limited. Our present study reveals that *GmGIF5*, the homolog of *AtGIF1*, regulates cell expansion rather than cell proliferation, offering new insights for the improvement of soybean molecular breeding.

There are eight identified members of the *GmGIF* gene family present in the soybean genome. Among them, *GmGIF5* exhibits the highest expression levels, particularly in meristematic tissues such as the shoot apical meristem and root tip (Figure 1). In this study, we generated *GmGIF5-*OE lines to examine the impact of *GmGIF5* on plant growth and development. After a 45-day cultivation period, we noted that the *GmGIF5*-OE lines exhibited increases in leaf area and leaf stalk length compared to those of the WT. Furthermore, the *GmGIF5*-OE lines displayed wider stems, taller plants, and bigger seeds than the WT (Figure 2, Figure 3 and Figure 4). Plant height, stem width, and leaf stalk length are components of plant architecture [1]. Bigger leaves during vegetative growth can contribute to higher photosynthesis ability and biomass accumulation, while a wider stem provides better support and transport ability [30]. Therefore, *GmGIF5* has the potential to positively promote plant biomass and yield. As *GIF*s have been widely reported to play a role in leaf size regulation [31,32,33] and in promoting cell proliferation [14,34], we further explored the underlying reasons for the enlargement of organs induced by *GmGIF5*. Histological analysis indicated that *GmGIF5* may regulate cell size to influence organ size. Based on our results, we conclude that *GmGIF5* mainly regulates cell size, although it may also play a role in regulating cell number. Given the high structural and functional conservation, it is possible that *GmGIF5* regulates both cell size and cell proliferation, likely in a cell type-dependent manner, ultimately influencing plant organ size and yield.

To further elucidate the mechanism underlying the influence of *GmGIF5* on plant growth and development, we performed RNA-seq. Although we identified only 123 DEGs, a principal component analysis and correlation analyses showed that our identification results were plausible (Figure S5). It is possible that *GmGIF5* only participates in certain pathways and influences several genes, resulting in a relatively small number of DEGs. Through function enrichment analysis, we found that the DEGs were significantly enriched in GO terms related to cell wall modification, mineral element metabolism, and photosynthesis. Most of the DEGs associated with cell wall modification processes, which are primarily involved in pectin metabolism, were downregulated in the *GmGIF5*-OE lines. Pectin regulates the permeability of the cell wall and exhibits structural variations throughout plant development. A reduced amount of pectin increases cell wall stiffness and reduces plasticity, thereby obstructing cell expansion [35,36,37,38]. Pectins can be degraded by pectinases, enzymes that are classified into various types based on their substrate specificity or mode of action, including pectin esterase, polygalacturonase, and pectate lyase [36]. Pectate lyase-like protein *AT3G01270* (*AtPLL10*), pectin methylesterases *AT2G43050* (*AtPME16*), *AT2G47040* (At*VGD1*), and *AT5G07430* (*AtPME50*), pectin acetylesterase *AT1G57590* (*AtPAE2*), and polygalacturonases *AT3G07850* and *AT3G14040* exhibited significantly downregulated expression in GmGIF5-overexpressing lines compared to in the wild type. Therefore, *GmGIF5* may regulate cell expansion by influencing cell wall strength through the modulation of pectin metabolism. In addition to genes related to pectin metabolism, other cell wall-associated genes also showed downregulated expression in the OE lines, such as *AT4G35010* (*AtBGAL11*). *AtBGAL11* encodes an enzyme that debranches galactan and influences cell wall loosening and cell expansion during pollen germination in Arabidopsis, and a recent study of apples indicated that *BGAL11* influences the flexibility and rigidity of apple cell layers [39,40].

Research shows that high levels of pectin methylesterification facilitate enhanced cell expansion, resource allocation to leaf growth, and increased leaf area for improved photosynthesis, collectively promoting enhanced plant growth [41,42]. Interestingly, the expression of genes that positively regulate photosynthesis was upregulated by *GmGIF5* overexpression, including *AT1G18400* (*AtBEE1*), *AT1G03010* (*NON-PHOTOTROPIC HYPOCOTYL 3*, *AtNPH3*) [43], *AT2G40100* (*AtLHCB4.3*), *AT3G47860* (*CHLOROPLASTIC LIPOCALIN*, *AtCHL*), and *AT1G68190* (*B-BOX DOMAIN PROTEIN 27*, *AtBBX27*). Notably, AtBEE1 was also found to interact with BBX28/29 to enhance brassinosteroid production and promote cell elongation-related genes, resulting in hypocotyl elongation [44]. What is more, iron is an important nutrient element required by soybeans and is closely related to their growth and yield [45]. The expression of genes related to iron homeostasis was also upregulated in the OE lines, namely, *AT1G76800* (*VACUOLAR IRON TRANSPORTER 2*, *AtVTL2*) [46] and *AT2G40300* (*FERRITIN 4*, *AtFER4*) [47]. The extracellular expression of ferritin in *Arabidopsis thaliana* can promote plant growth and iron accumulation [48]. Overall, the RNA-seq results indicated that *GmGIF5* regulates cell modification genes and brassinosteroid-related genes to regulate pectin metabolism and promote cell expansion. Simultaneously, *GmGIF5* enhances photosynthesis and iron assimilation for biomass accumulation.

## 4. Materials and Methods

### 4.1. Plant Material and Phenotype Data Collection

The wild-type and background Arabidopsis plants used as the materials in this study were of the Columbia-0 (Col-0) ecotype. Arabidopsis seeds were vernalized at 4 °C for two days before being sowed on 1/2 Murashige and Skoog plates. Eight days later, the seedlings were transplanted into experimental plots. To construct *GmGIF5* overexpression (OE) vectors, we used the pCAMBIA 1300 plant expression vector containing the constitutive promoter *pUBQ10* [49]. The primers are listed in Table S6. Transgenic lines were obtained using the floral-dip method [50]. Glyphosate-resistant T_3_ generation lines were detected at the DNA and mRNA levels. The generation of a 1 cm bolting from the rosette was considered to indicate flowering. Leaf size and leaf epidermis cell size were measured at 45 days after transplantation, whereas plant height, stem width, and seed phenotype were measured at 75 days after transplantation. For phenotype collection, 12 plants of each OE line and the wild type (WT) were used. Statistical significance analyses were performed by Excel 2019, and dot plots were constructed using the R package ggplot2 (ver 3.4.2) [51] in R studio (ver 4.3.1).

### 4.2. Bioinformatic Analysis

Subcellular localization prediction was conducted using the WoLF PSORT tool [52] (https://wolfpsort.hgc.jp, accessed on 12 September 2023), while the ExPASy [53] website tool (http://www.expasy.org, accessed on 12 September 2023) was applied to predict the molecular weight, isoelectric point, and the grand average of hydropathicity (Table S1).

The protein sequences of *Arabidopsis thaliana* (version: Athaliana_447_Araport11), *Arachis hypogaea* (version: v 1.0), *Oryza sativa* (version: v 7.0), *Zea mays* (version: v 4), *Gossypium hirsutum* (version: v 3.1), and *Glycine max* (version: Wm82.a4.v1) were downloaded from the Phytozome database [54] (https://phytozome-next.jgi.doe.gov/, accessed on 14 September 2023). The protein sequences of *Brassica napus* were downloaded from BnIR [55] (https://yanglab.hzau.edu.cn/BnIR/, accessed on 14 September 2023). To identify homologous genes in different plants, a hidden Markov model (HMM) of the SSXT domain was applied in the Pfam database [56] (ACC: PF05030), and the HMMsearch (version 3.0) tool [57] was used to screen sequences with the SSXT domain. All the putative GIF proteins were checked in the National Center for Biotechnology Information (NCBI) conserved domain database (CDD) (https://www.ncbi.nlm.nih.gov/cdd, accessed on 15 September 2023). All the verified GIF protein sequences were aligned using MAFFT (version 7.380) [58], and a phylogenetic tree using a BLOSUM62 model was constructed based on the alignment results using Fast Tree (version 2.1.7) and MEGA 7 [59] with maximum-likelihood estimation based on the most suitable model and 1000 bootstraps. The Evolview online tool [60] was used to embellish the phylogenetic tree.

### 4.3. RNA Extraction and Reverse Transcription–Quantitative Polymerase Chain Reaction (RT-qPCR) Analysis

Full ‘Tianlong No. 1′ soybean seeds were selected and placed in nutrient soil and cultivated in a cultivation room (light cycle of 16 h/8 h, light intensity of 300–400 μmol·m^−2^·s^−1^, and temperature of 25 ± 2 °C) with watering at intervals to ensure normal growth. The total RNA of soybean tissues (flower, shoot, shoot tip, leaf, seed, nodule, and pods) was extracted using the TransZol Up Plus RNA Kit (Transgene, Beijing, China). First-strand synthesis was performed using Hifair^®^ III 1st Strand cDNA Synthesis SuperMix for qPCR (gDNA digester plus) (YEASON, Shanghai, China) following the manufacturer’s protocol.

TB Green Premix Ex Taq^TM^ (Tli RNaseH Plus) (Takara, Tokyo, Japan) was used for RT-qPCR analysis following the manufacturer’s protocol. *GmCons4* was used as a reference gene for normalization [61]. Primers are listed in Table S6. Three individual replicate reactions were run for each gene. The relative expression was calculated using 2^−ΔCT^ quantitative analysis. Bar plots were constructed with Excel 2019.

### 4.4. Subcellular Localization

pCAMBIA1305-GFP [29] was linearized by *Spe*1 (Takara, Tokyo, Japan) for 1 h at 37 °C. The *GmGIF5* coding sequence without the stop codon was amplified and fused with a linearized pCAMBIA1305-GFP vector using the In-Fusion^®^ HD Cloning Kit (Takara, Tokyo, Japan). The obtained fusion product was first transformed into *Escherichia coli* strain DH5α purchased from YEASON (Shanghai, China). The constructed effector plasmids were transformed into *Agrobacterium tumefaciens* strain GV3101 (pSoup19) and then transiently transformed into *Nicotiana benthamiana* leaves. OsD53-mCherry was used as a nucleus marker [62]. After 48 h, the leaves were collected and observed under a laser-scanning confocal microscope (LSM980 laser, Carl Zeiss, Oberkochen, Germany). An empty vector served as the negative control in this experiment. Green fluorescent protein (GFP) signals were recorded with an excitation wavelength of 488 nm and an emission wavelength ranging from 505 to 530 nm [63].

### 4.5. Transcriptional Activity Assay

The whole coding sequence was amplified and incorporated into a linearized pGBKT7 vector [64] to construct the fusion vector BD-GmGIF5. The verified fusion plasmids and the negative control BD were transferred into *Saccharomyces cerevisiae* strain AH109 (YEASON, Shanghai, China), respectively. Positive colonies were grown on SD/-Trp to confirm the presence of transgenes, whereas transformed cells were grown on SD/-His/-Leu/-Trp to show activation ability. GmNAC181 was used as a positive control [64].

### 4.6. RNA-Seq and Transcriptome Validation

For the RNA-seq assay, aerial parts from the OE2 and WT lines were collected 25 days after transplantation. Samples were immediately frozen in liquid nitrogen and stored at –80 °C until processing and analysis. RNA extraction and cDNA library construction were performed using the TransZol Up Plus RNA Kit (Transgene, Beijing, China) and Hifair^®^ III 1st Strand cDNA Synthesis SuperMix for qPCR (gDNA digester plus) (YEASON, Shanghai, China), respectively, following the manufacturers’ protocols. Raw data were generated after sequencing using the Illumina NovaSeq platform, and clean data were obtained after filtering. The data filtering criteria are as follows: (1) Reads with N base contents exceeding 5% are removed. (2) Reads with low-quality (a quality value smaller than 5) bases accounting for 50% or more are removed. (3) Reads contaminated with adapters are removed. (4) Reads resulting from PCR amplification and causing duplicates are removed. (5) Reads with lengths less than 30 bp are filtered out. Clean data were mapped onto the reference genome using Star (version 2.7.9a) [65] with default parameters. Sequencing data were deposited in NCBI Bioproject under ID PRJNA1121023. Three biological replicates were used in the RNA-seq experiment. RSEM (version 1.3.3) [66] was used to convert gene counts into fragments per kilobase per million bases (FPKM). Sample correlations were calculated and visualized by TBtools-II (version 2.142) [67], and PCA was performed and visualized by the R package ggplot2 (ver 3.4.2) [51]. Differentially expressed genes (DEGs) were identified using DEseq2 (version 1.26.0) [68] according to a threshold of *p* < 0.05 and log_2_|fold change| > 1. ClusterProfiler (version 3.14.3) was applied to perform the functional enrichment of DEGs [69]; GO terms and KEGG pathways with FDR < 0.05 were selected.

*AtActin2* was used as an internal reference gene for RT-qPCR. All primers used are listed in Table S6. The relative expression levels were calculated using the 2^−ΔCt^ method; significance analysis and visualization were conducted in Excel 2019.

## 5. Conclusions

In this study, we demonstrated that the nuclear protein GmGIF5 has self-transactivation activity and plays a vital role in plant growth and development. The overexpression of *GmGIF5* in Arabidopsis resulted in significant increases in leaf area, plant height, stem thickness, and thousand-grain weight. Cytological observations and RNA-seq analysis revealed that the overexpression of *GmGIF5* promotes plant cell expansion by regulating cell wall modifications, thereby affecting organ size and increasing biomass accumulation. Reports indicate that *GIFs* are involved in cell proliferation in many species. Our research shows that *GmGIF5* can regulate cell expansion, providing new insights into the biological functions of *GIF* genes and new directions for soybean breeding.

## Data Availability

The datasets presented in this study can be found in online repositories. The names of the repository/repositories and the accession number can be found below: NCBI SRA BioProject, accession no: PRJNA1121023, https://dataview.ncbi.nlm.nih.gov/object/PRJNA1121023?reviewer=hfdr4h0ttetvkd4svgdapcvj6t (accessed on 12 November 2024).

## Supplementary Materials

The following supporting information can be downloaded at https://www.mdpi.com/article/10.3390/ijms26020492/s1.

## References

[1] Liu S., Zhang M., Feng F., Tian Z. (2020). Toward a “Green Revolution” for Soybean. Mol. Plant.

[2] Machado F.B., Moharana K.C., Almeida-Silva F., Gazara R.K., Pedrosa-Silva F., Coelho F.S., Grativol C., Venancio T.M. (2020). Systematic Analysis of 1298 RNA-seq Samples and Construction of a Comprehensive Soybean (*Glycine max*) Expression Atlas. Plant J..

[3] Yu T.F., Hou Z.H., Wang H.L., Chang S.Y., Song X.Y., Zheng W.J., Zheng L., Wei J.T., Lu Z.W., Chen J. (2024). Soybean Steroids Improve Crop Abiotic Stress Tolerance and Increase Yield. Plant Biotechnol. J..

[4] Chen L., Yang H., Fang Y., Guo W., Chen H., Zhang X., Dai W., Chen S., Hao Q., Yuan S. (2021). Overexpression of GmMYB14 Improves High-Density Yield and Drought Tolerance of Soybean through Regulating Plant Architecture Mediated by the Brassinosteroid Pathway. Plant Biotechnol. J..

[5] Wang H.W., Zhang B., Hao Y.J., Huang J., Tian A.G., Liao Y., Zhang J.S., Chen S.Y. (2007). The Soybean Dof-Type Transcription Factor Genes, *GmDof4* and *GmDof11*, Enhance Lipid Content in the Seeds of Transgenic *Arabidopsis* Plants. Plant J..

[6] Lyu J., Wang D., Sun N., Yang F., Li X., Mu J., Zhou R., Zheng G., Yang X., Zhang C. (2024). The *TaSnRK1-TabHLH489* Module Integrates Brassinosteroid and Sugar Signalling to Regulate the Grain Length in Bread Wheat. Plant Biotechnol. J..

[7] Li S., Gao F., Xie K., Zeng X., Cao Y., Zeng J., He Z., Ren Y., Li W., Deng Q. (2016). The OsmiR396c-OsGRF4-OsGIF1 Regulatory Module Determines Grain Size and Yield in Rice. Plant Biotechnol. J..

[8] Liu H., Guo S., Xu Y., Li C., Zhang Z., Zhang D., Xu S., Zhang C., Chong K. (2014). OsmiR396d-Regulated OsGRFs Function in Floral Organogenesis in Rice through Binding to Their Targets *OsJMJ706* and *OsCR4*. Plant Physiol..

[9] Zhang Y., Mo Y., Ren H., Wu X., Han L., Sun Z., Xu W. (2024). Improving Sedum Plumbizincicola Genetic Transformation with the *SpGRF4–SpGIF1* Gene and the Self-Excision CRE/LoxP System. Planta.

[10] Hussain E., Es Romanowski A., Halliday K.J. (2022). *PIF7* Controls Leaf Cell Proliferation through an AN3 Substitution Repression Mechanism. Proc. Natl. Acad. Sci. USA.

[11] Ercoli M.F., Ferela A., Debernardi J.M., Perrone A.P., Rodriguez R.E., Palatnik J.F. (2018). GIF Transcriptional Coregulators Control Root Meristem Homeostasis. Plant Cell.

[12] Horiguchi G., Kim G.T., Tsukaya H. (2005). The Transcription Factor AtGRF5 and the Transcription Coactivator AN3 Regulate Cell Proliferation in Leaf Primordia of *Arabidopsis thaliana*. Plant J..

[13] Liu Z., Li N., Zhang Y., Li Y. (2020). Transcriptional Repression of GIF1 by the KIX-PPD-MYC Repressor Complex Controls Seed Size in *Arabidopsis*. Nat. Commun..

[14] Ezaki K., Koga H., Takeda-Kamiya N., Toyooka K., Higaki T., Sakamoto S., Tsukaya H. (2024). Precocious Cell Differentiation Occurs in Proliferating Cells in Leaf Primordia in *Arabidopsis angustifolia3* Mutant. Front. Plant Sci..

[15] He Z., Zeng J., Ren Y., Chen D., Li W., Gao F., Cao Y., Luo T., Yuan G., Wu X. (2017). *OsGIF1* Positively Regulates the Sizes of Stems, Leaves, and Grains in Rice. Front. Plant Sci..

[16] Kim J.H. (2019). Biological Roles and an Evolutionary Sketch of the GRF-GIF Transcriptional Complex in Plants. BMB Rep..

[17] Wang H., Sha G., Gao R., Pang J., Zhai R., Yang C., Wang Z., Xu L. (2024). *PbGIF1* Promoting Cell-Proliferation in Pear Fruit Is Transcriptionally Activated by *PbRR1*. Hortic. Plant J..

[18] Alam I., Wu X., Ge L. (2022). Comprehensive Genomic Survey, Evolution, and Expression Analysis of GIF Gene Family during the Development and Metal Ion Stress Responses in Soybean. Plants.

[19] Cosgrove D.J. (2005). Growth of the Plant Cell Wall. Nat. Rev. Mol. Cell Biol..

[20] Bonnin E., Garnier C., Ralet M.C. (2014). Pectin-Modifying Enzymes and Pectin-Derived Materials: Applications and Impacts. Appl. Microbiol. Biotechnol..

[21] Jiang J., Jiang J., Qiu L., Miao Y., Yao L., Cao J. (2013). Identification of gene expression profile during fertilization in *Brassica campestris subsp. chinensis*. Genome.

[22] Jiang L., Yang S.L., Xie L.F., Puah C.S., Zhang X.Q., Yang W.C., Sundaresan V., Ye D. (2005). *VANGUARD1* Encodes a Pectin Methylesterase That Enhances Pollen Tube Growth in the *Arabidopsis* Style and Transmitting Tract. Plant Cell.

[23] Tian G.W., Chen M.H., Zaltsman A., Citovsky V. (2006). Pollen-Specific Pectin Methylesterase Involved in Pollen Tube Growth. Dev. Biol..

[24] Philippe F., Pelloux J., Rayon C. (2017). Plant Pectin Acetylesterase Structure and Function: New Insights from Bioinformatic Analysis. BMC Genom..

[25] Mollet J.C., Leroux C., Dardelle F., Lehner A. (2013). Cell Wall Composition, Biosynthesis and Remodeling during Pollen Tube Growth. Plants.

[26] Mabuchi A., Soga K., Wakabayashi K., Hoson T. (2016). Phenotypic Screening of Arabidopsis T-DNA Insertion Lines for Cell Wall Mechanical Properties Revealed *ANTHOCYANINLESS2*, a Cell Wall-Related Gene. J. Plant Physiol..

[27] Zhong R., Peña M.J., Zhou G.K., Nairn C.J., Wood-Jones A., Richardson E.A., Morrison W.H., Darvill A.G., York W.S., Ye Z.H. (2005). *Arabidopsis Fragile Fiber8*, Which Encodes a Putative Glucuronyltransferase, Is Essential for Normal Secondary Wall Synthesis. Plant Cell.

[28] Wang F., Gao Y., Liu Y., Zhang X., Gu X., Ma D., Zhao Z., Yuan Z., Xue H., Liu H. (2019). BES1-Regulated BEE1 Controls Photoperiodic Flowering Downstream of Blue Light Signaling Pathway in *Arabidopsis*. New Phytol..

[29] Zhou C., Lin Q., Ren Y., Lan J., Miao R., Feng M., Wang X., Liu X., Zhang S., Pan T. (2023). A CYP78As–Small Grain4–Coat Protein Complex Ⅱ Pathway Promotes Grain Size in Rice. Plant Cell.

[30] Zhu X.G., Long S.P., Ort D.R. (2010). Improving Photosynthetic Efficiency for Greater Yield. Annu. Rev. Plant Biol..

[31] Lee B.H., Ko J.H., Lee S., Lee Y., Pak J.H., Kim J.H. (2009). The *Arabidopsis* GRF-Interacting Factor Gene Family Performs an Overlapping Function in Determining Organ Size as Well as Multiple. Plant Physiol..

[32] Lee B.H., Wynn A.N., Franks R.G., Hwang Y., Lim J., Kim J.H. (2014). The *Arabidopsis thaliana* GRF-Interacting Factor Gene Family Plays an Essential Role in Control of Male and Female Reproductive Development. Dev. Biol..

[33] Lee B.H., Kim J.H. (2014). Spatio-Temporal Distribution Patterns of *GRF-INTERACTING FACTOR* Expression and Leaf Size Control. Plant Signal Behav..

[34] Jun S.E., Kim J.H., Hwang J.Y., Huynh Le T.T., Kim G.T. (2020). ORESARA15 Acts Synergistically with ANGUSTISFOLIA3 and Separately from AINTEGUMENTA to Promote Cell Proliferation during Leaf Growth. Int. J. Mol. Sci..

[35] Wu H.C., Bulgakov V.P., Jinn T.L. (2018). Pectin Methylesterases: Cell Wall Remodeling Proteins Are Required for Plant Response to Heat Stress. Front. Plant Sci..

[36] Cao J. (2012). The Pectin Lyases in *Arabidopsis thaliana*: Evolution, Selection and Expression Profiles. PLoS ONE.

[37] Haas K.T., Wightman R., Peaucelle A., Höfte H. (2021). The Role of Pectin Phase Separation in Plant Cell Wall Assembly and Growth. Cell Surf..

[38] Xiong J., Yang Y., Fu G., Tao L. (2015). Novel Roles of Hydrogen Peroxide (H_2_O_2_) in Regulating Pectin Synthesis and Demethylesterification in the Cell Wall of Rice (*Oryza Sativa*) Root Tips. New Phyto.

[39] Collins P.P., O’donoghue E.M., Rebstock R., Tiffin H.R., Sutherland P.W., Schröder R., Mcatee P.A., Prakash R., Ireland H.S., Johnston J.W. (2019). Cell Type-Specific Gene Expression Underpins Remodeling of Cell Wall Pectin in Exocarp and Cortex during Apple Fruit Development. J. Exp. Bot..

[40] Hrubá P., Honys D., Twell D., Čapková V., Tupý J. (2005). Expression of *β-*Galactosidase and *β-*Xylosidase Genes during Microspore and Pollen Development. Planta.

[41] Kim S.J., Held M.A., Zemelis S., Wilkerson C., Brandizzi F. (2015). CGR2 and CGR3 Have Critical Overlapping Roles in Pectin Methylesterification and Plant Growth in *Arabidopsis thaliana*. Plant J..

[42] Weraduwage S.M., Kim S.J., Renna L., Anozie F.C., Sharkey T.D., Brandizzi F. (2016). Pectin Methylesterification Impacts the Relationship between Photosynthesis and Plant Growth. Plant Physiol..

[43] Molas M.L., Kiss J.Z., Correll M.J. (2006). Gene Profiling of the Red Light Signaling Pathways in Roots. J. Exp. Bot..

[44] Cao J., Liang Y., Yan T., Wang X., Zhou H., Chen C., Zhang Y., Zhang B., Zhang S., Liao J. (2022). The Photomorphogenic Repressors BBX28 and BBX29 Integrate Light and Brassinosteroid Signaling to Inhibit Seedling Development in *Arabidopsis*. Plant Cell.

[45] Chu Q., Sha Z., Maruyama H., Yang L., Pan G., Xue L., Watanabe T. (2019). Metabolic Reprogramming in Nodules, Roots, and Leaves of Symbiotic Soybean in Response to Iron Deficiency. Plant Cell Environ..

[46] Gollhofer J., Timofeev R., Lan P., Schmidt W., Buckhout T.J. (2014). Vacuolar-Iron-Transporter1-Like Proteins Mediate Iron Homeostasis in *Arabidopsis*. PLoS ONE.

[47] Murgia I., Vigani G. (2015). Analysis of *Arabidopsis thaliana atfer4-1, atfh* and *atfer4-1/atfh* Mutants Uncovers Frataxin and Ferritin Contributions to Leaf Ionome Homeostasis. Plant Physiol. Biochem..

[48] Lin C.Y., Jakes J.E., Donohoe B.S., Ciesielski P.N., Yang H., Gleber S.C., Vogt S., Ding S.Y., Peer W.A., Murphy A.S. (2016). Directed Plant Cell-Wall Accumulation of Iron: Embedding Co-Catalyst for Efficient Biomass Conversion. Biotechnol. Biofuels.

[49] Zhan Y., Wu T., Zhao X., Wang J., Guo S., Chen S., Qu S., Zheng Z. (2023). Genome-Wide Identification and Expression of Monoacylglycerol Lipase (MAGL) Gene Family in Peanut (*Arachis hypogaea* L.) and Functional Analysis of AhMGATs in Neutral Lipid Metabolism. Int. J. Biol. Macromol..

[50] Clough S.J., Bent A.F. (1998). Floral Dip: A Simplified Method for Agrobacterium-Mediated Transformation of *Arabidopsis thaliana*. Plant J..

[51] Valero-Mora P.M. (2010). ggplot2: Elegant Graphics for Data Analysis. J. Stat. Softw..

[52] Horton P., Park K.J., Obayashi T., Fujita N., Harada H., Adams-Collier C.J., Nakai K. (2007). WoLF PSORT: Protein Localization Predictor. Nucleic Acids Res..

[53] Artimo P., Jonnalagedda M., Arnold K., Baratin D., Csardi G., De Castro E., Duvaud S., Flegel V., Fortier A., Gasteiger E. (2012). ExPASy: SIB Bioinformatics Resource Portal. Nucleic Acids Res..

[54] Goodstein D.M., Shu S., Howson R., Neupane R., Hayes R.D., Fazo J., Mitros T., Dirks W., Hellsten U., Putnam N. (2012). Phytozome: A Comparative Platform for Green Plant Genomics. Nucleic Acids Res..

[55] Yang Z., Wang S., Wei L., Huang Y., Liu D., Jia Y., Luo C., Lin Y., Liang C., Hu Y. (2023). BnIR: A Multi-Omics Database with Various Tools for Brassica Napus Research and Breeding. Mol. Plant.

[56] Mistry J., Chuguransky S., Williams L., Qureshi M., Salazar G.A., Sonnhammer E.L.L., Tosatto S.C.E., Paladin L., Raj S., Richardson L.J. (2021). Pfam: The Protein Families Database in 2021. Nucleic Acids Res.

[57] Potter S.C., Luciani A., Eddy S.R., Park Y., Lopez R., Finn R.D. (2018). HMMER Web Server: 2018 Update. Nucleic Acids Res..

[58] Katoh K., Misawa K., Kuma K.I., Miyata T. (2002). MAFFT: A Novel Method for Rapid Multiple Sequence Alignment Based on Fast Fourier Transform. Nucleic Acids Res..

[59] Kumar S., Stecher G., Tamura K. (2016). MEGA7: Molecular Evolutionary Genetics Analysis Version 7.0 for Bigger Datasets. Mol. Biol. Evol..

[60] Subramanian B., Gao S., Lercher M.J., Hu S., Chen W.H. (2019). Evolview v3: A Webserver for Visualization, Annotation, and Management of Phylogenetic Trees. Nucleic Acids Res..

[61] Wei Z., Pan T., Zhao Y., Su B., Ren Y., Qiu L. (2020). Rab5a and Its GEFs Are Involved in Post-Golgi Trafficking of Storage Proteins in Developing Soybean Cotyledon. J. Exp. Bot..

[62] Zhou F., Lin Q., Zhu L., Ren Y., Zhou K., Shabek N., Wu F., Mao H., Dong W., Gan L. (2013). D14–SCFD3-Dependent Degradation of D53 Regulates Strigolactone Signaling. Nature.

[63] Bao X., Wang Y., Qi Y., Lei C., Wang Y., Pan T., Yu M., Zhang Y., Wu H., Zhang P. (2023). A Deleterious Sar1c Variant in Rice Inhibits Export of Seed Storage Proteins from the Endoplasmic Reticulum. Plant Mol. Biol..

[64] Wang X., Chen K., Zhou M., Gao Y., Huang H., Liu C., Fan Y., Fan Z., Wang Y., Li X. (2022). GmNAC181 Promotes Symbiotic Nodulation and Salt Tolerance of Nodulation by Directly Regulating *GmNINa* Expression in Soybean. New Phytol..

[65] Dobin A., Davis C.A., Schlesinger F., Drenkow J., Zaleski C., Jha S., Batut P., Chaisson M., Gingeras T.R. (2013). STAR: Ultrafast Universal RNA-seq Aligner. Bioinformatics.

[66] Li B., Dewey C.N. (2011). RSEM: Accurate Transcript Quantification from RNA-seq Data with or without a Reference Genome. BMC Bioinform..

[67] Chen C., Wu Y., Li J., Wang X., Zeng Z., Xu J., Liu Y., Feng J., Chen H., He Y. (2023). TBtools-II: A “One for All, All for One” Bioinformatics Platform for Biological Big-Data Mining. Mol. Plant.

[68] Love M.I., Huber W., Anders S. (2014). Moderated Estimation of Fold Change and Dispersion for RNA-seq Data with DESeq2. Genome Biol..

[69] Yu G., Wang L.G., Han Y., He Q.Y. (2012). ClusterProfiler: An R Package for Comparing Biological Themes among Gene Clusters. OMICS.

