# Supplementary material for: GmGIF5 Promotes Cell Expansion by Negatively Regulating Cell Wall Modification
Source: Int J Mol Sci. 2025 Jan 9;26(2):492. doi: 10.3390/ijms26020492 (PMC11765367; doi:10.3390/ijms26020492)
Supplement: Supplementary file 1 [file ijms-26-00492-s001.zip › Supplemental Figure_Revision.pdf]

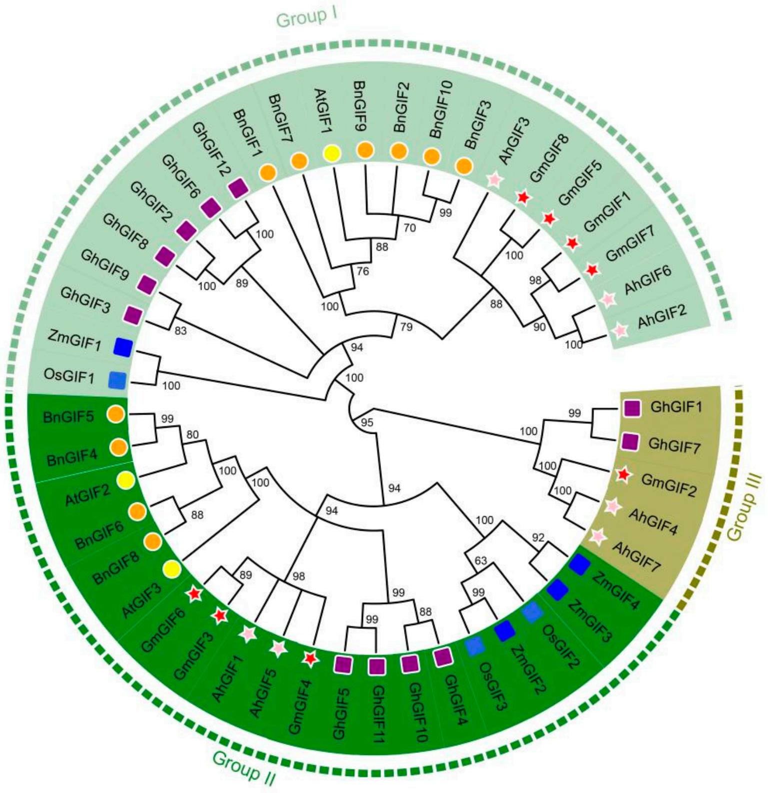

Figure S1. Phylogenetic relationship analysis of GmGIF proteins. Phylogenetic tree of *A. thaliana*, *A. hypogaea*, *O. sativa*, *Z. mays*, *Br. napus*, *G. hirsutum*, and *G. max* GIF proteins. The maximum-likelihood (ML) phylogenetic tree was generated by MEGA7. Yellow and orange circles denote AtGIF and BnGIF proteins, respectively. GhGIF proteins are represented by purple triangles. Bule and dark-blue cubes indicate OsGIF and ZmGIF proteins, respectively. AhGIF proteins are featured by pink stars and GmGIFs are indicated by red stars. The bootstrap values are 1000. Protein IDs of the analyzed Proteins can be found in Table S2.

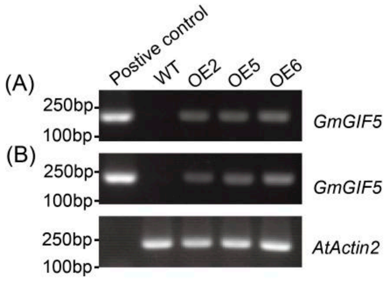

Figure S2. Validation of *GmGIF5* at the DNA and RNA levels in *GmGIF5* overexpression lines. (A) DNA and (B) mRNA levels of *GmGIF5* in wild-type (WT) and overexpression lines. *AtActin2* was used as an internal control. The *GmGIF5*-overexpression vector was used as a positive control.

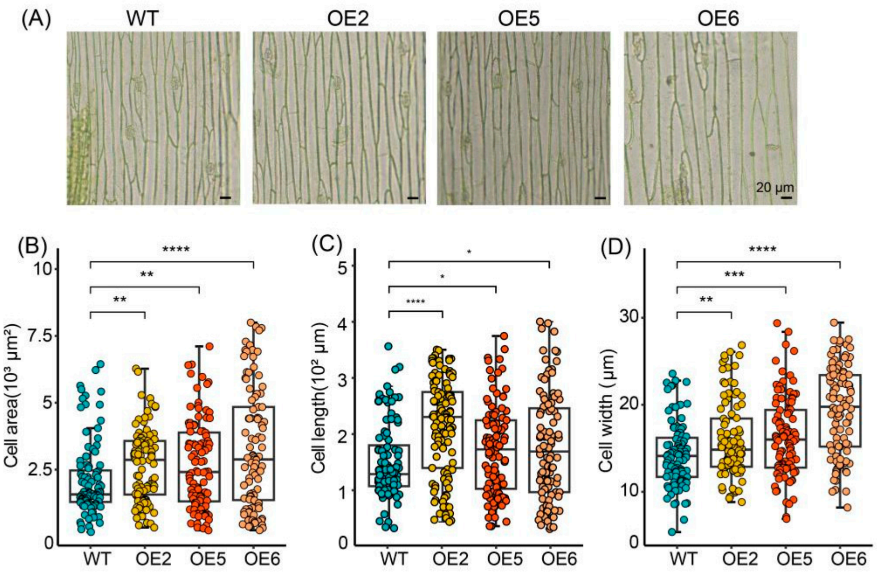

Figure S3. Overexpression of *GmGIF5* increased stem cell size in Arabidopsis. (A) Epidermal cell observation from wild-type and transgenic Arabidopsis at 35 days after transplantation. (B-D) Measurement of cell area (B), cell length (C), and cell width (D) of the epidermal cells. Boxes represent the median values, the first, and third quartiles; whiskers represent the minimum and maximum values. The dots represent single values in boxplots. *$p < 0.05$, **$p < 0.01$, ***$p < 0.001$, and ****$p < 0.0001$ (Student's *t*-test).

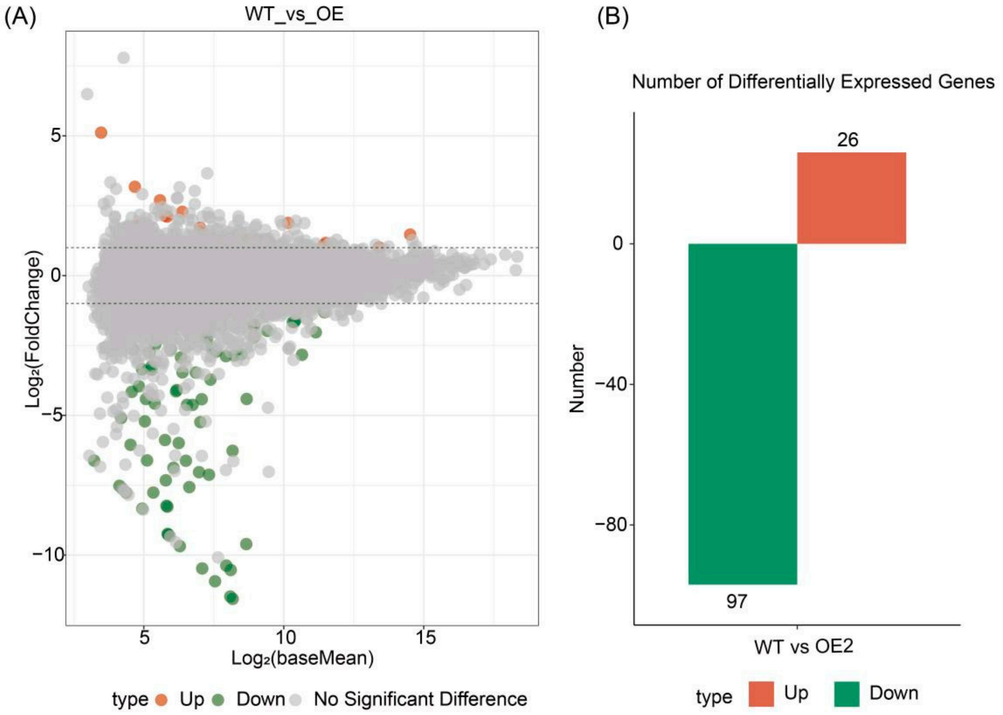

Figure S4. Features of differentially expressed genes between overexpression 2 (OE2) and wild-type (WT) lines. (A) Dot plot showing gene expression profiles of OE2 and WT lines. (B) Among the identified

differentially expressed genes, 97 were down-regulated (green) and 26 were up-regulated (red) in OE2 compared to the WT.

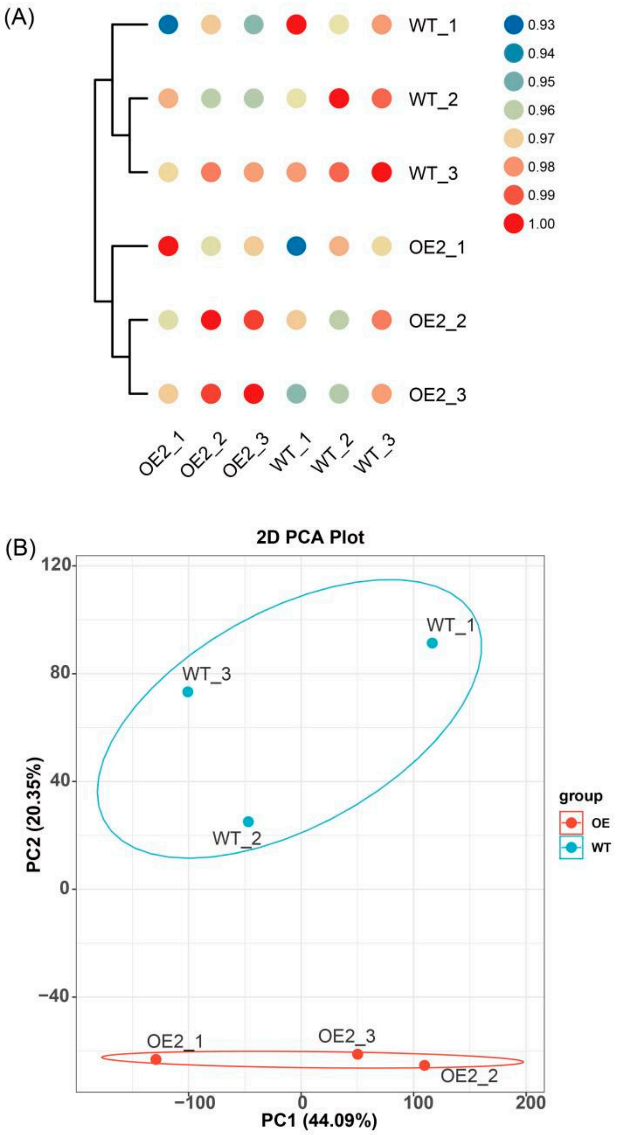

Figure S5. Sample correlation analysis. (A) Correlation analysis of samples with Pearson's correlation coefficient. (B) Principal component analysis of samples. Red dots represent relative higher correlation between samples, blue dots represent lower correlation. Overexpression (OE) samples are represented by red dots and wild-type (WT) samples are represented by blue dots.
